# How common is germinal mosaicism that leads to premeiotic aneuploidy in the female?

**DOI:** 10.1007/s10815-019-01596-6

**Published:** 2019-11-08

**Authors:** Joy DA Delhanty, Sioban B SenGupta, Harita Ghevaria

**Affiliations:** Preimplantation Genetics Group, Institute for Women’s Health, University College London, 86-96 Chenies Mews, London, WC1E 6HX U.K.

**Keywords:** Premeiotic aneuploidy, Germinal mosaicism, Metaphase II oocyte and first PB complex, FISH, CGH, Meiosis, Trisomy 21

## Abstract

**Purpose:**

Molecular cytogenetic analysis has confirmed that a proportion of apparently meiotic aneuploidy may be present in the germ cells prior to the onset of meiosis, but there is no clear perception of its frequency. The aim of this review is to assess the evidence for premeiotic aneuploidy from a variety of sources to arrive at an estimate of its overall contribution to oocyte aneuploidy in humans.

**Methods:**

Relevant scientific literature was covered from 1985 to 2018 by searching PubMed databases with search terms: gonadal/germinal mosaicism, ovarian mosaicism, premeiotic aneuploidy, meiosis and trisomy 21. Additionally, a key reference from 1966 was included.

**Results:**

Data from over 9000 cases of Down syndrome showed a bimodal maternal age distribution curve, indicating two overlapping distributions. One of these matched the pattern for the control population, with a peak at about 28 years and included all cases that had occurred independently of maternal age, including those due to germinal mosaicism, about 40% of the cohort. The first cytological proof of germinal mosaicism was obtained by fluorescence in situ hybridisation analysis. Comparative genomic hybridisation analysis of oocyte chromosomes suggests an incidence of up to 15% in premeiotic oocytes. Direct investigation of fetal ovarian cells led to variable results for chromosome 21 mosaicism.

**Conclusions:**

Oocytes with premeiotic errors will significantly contribute to the high level of preimplantation and prenatal death. Data so far available suggests that, depending upon the maternal age, up to 40% of aneuploidy that is present in oocytes at the end of meiosis I may be due to germinal mosaicism.

## Introduction

Aneuploidy is the most common cause of death in the human population from the moment of conception onwards. While the rare existence of gonadal mosaicism is well recognised, it is generally assumed that aneuploidy has its origins during the first or second division of meiosis and in the case of the autosomes that this is mainly confined to oogenesis. The advent and further development of techniques of molecular cytogenetics such as fluorescence *in situ* hybridisation (FISH), comparative genomic hybridisation (CGH), microarray CGH and next generation sequencing (NGS), in recent decades has allowed an in-depth analysis of events during male and female meiosis and has directly confirmed that very high levels of aneuploidy occur in oocytes of women at the end of their reproductive life [1, 2]. However, the application of these technical developments has also shown that an unknown proportion of apparently meiotic aneuploidy may in fact already be present in the germ cells prior to the onset of meiosis [3–5]. Similarly, molecular cytogenetic analyses of cells from the human preimplantation embryo have demonstrated the extraordinarily high frequency of mosaic aneuploidy, of mitotic origin, at this stage of early human life [2, 6–9]. Together, meiotic and post-zygotic aneuploidy results in the demise of three quarters of embryos conceived by in vitro fertilisation (IVF) before implantation can take place [10]. Surviving embryos with low level mosaicism may be eliminated by spontaneous abortion in very early pregnancy; of those exhibiting abnormal karyotypes in uncultured tissue, mosaicism has been detected in almost 50% by detailed interphase FISH analysis [11]. Other mosaic embryos may result in the birth of healthy individuals that carry a low percentage of aneuploid cells in somatic and/or germline cells. A link has been proposed between somatic mosaicism and the development of certain cancers [12] while germinal mosaicism will predispose to premeiotic aneuploidy. This review will consider the evidence concerning premeiotic aneuploidy in the female.

## Premeiotic aneuploidy

The timing of the post-zygotic error leading to chromosomal mosaicism will clearly affect whether the abnormal cell line is widespread or just confined to a single-cell lineage. Errors may of course occur in any one of the estimated 10^16^ mitotic cell divisions required to form an adult human, but in cases where the mosaicism has been confirmed in both lymphocytes and epithelial tissue, the origin must predate the differentiation of the distinct cell lines in the embryo that occurs during days 4–7 of embryonic development.

Mosaicism confined to the germline is difficult to detect. There are two types: gonadal mosaicism or germinal/germline mosaicism. Where there is evidence of repeated conceptions with the same chromosomal anomaly involved in each case, then it may be assumed that the primordial germ cells are affected and the term gonadal mosaicism is appropriate [3]. In the second week post-conception, the primordial germ cells, consisting of about 100 cells, migrate to the hind gut. By week 5, the number of oogonia lies between 5000 and 7000, but by weeks 19–20, there is a total of about 5 million [13, 14]. This enormous increase in cell number provides ample opportunity for the occurrence of mitotic errors that may affect only a single mature oocyte. Where it is not known exactly when the abnormal event took place, except that it is premeiotic, we have suggested the term germinal or germline mosaicism [5, 15]. It is important to understand that aneuploidy that derives from errors in mitosis in the oogonia will not be detectable in post-natal karyotype analysis of somatic tissues. Mosaicism detected by routine karyotype analysis in families with multiple aneuploid conceptions will therefore always give an underestimate of the true rate of occurrence of that which extends to the germline.

### Parental mosaicism in Down syndrome

Recurrent miscarriage or abnormal birth due to repeated occurrence of the same trisomy or different trisomies may be due to chance [16] or due to demonstrable parental mosaicism affecting somatic cells [17]. It is inevitable, since Down syndrome (DS) is the only clinical autosomal syndrome with a large number of survivors, that the following data analyses concentrate on trisomy 21, but there is no reason to suppose that the principles will not apply to other trisomy syndromes. By the early 1980s, there had been many case reports of parental mosaicism in families with more than one child affected with DS. Uchida and Freeman set out to investigate a random series of 374 families with the aim of identifying the frequency of parental mosaicism [18]. From the parents, at least 100 lymphocyte metaphases were analysed; in 10 of the 374 (2.7%) families, a parent had two or more trisomic cells. In seven cases, the mother was the mosaic, and in the remainder, it was the father. Interestingly, in one mother where the stroma of the ovaries was available for analysis, the frequency of trisomic cells in lymphocytes and skin fibroblasts was 7.4% and 2%, respectively, whereas in the right and left ovaries, it was 24% and 22% [18]. More selectively, Sachs and co-workers followed up 1211 pregnancies that occurred after the birth of a child with trisomy 21 (T21); in the great majority, the women were over the age of 36 at the time [19]. There were six instances of T21 in the subsequent pregnancy. Chromosomes of parental lymphocytes, fibroblasts and, in one case, ovaries were studied and mosaicism was detected in two couples that had had three and four affected pregnancies. In one case, the father had normal lymphocyte chromosomes but 22% of T21 cells in fibroblasts; in the other case the mother’s lymphocytes were 3% trisomic, fibroblasts 14% and left and right ovaries 47% and 44%, respectively [19]. Families with known sibling recurrence of DS were investigated cytogenetically by two groups. Pangalos et al. found parental mosaicism in 5 of 13 families; in one, it was the father, and in the other four, it was the mother that was mosaic. In the maternal cases, the ages at the birth of the first DS child were 26, 28, 25 and 36 years [20]. James and colleagues investigated four families each of which had three T21 pregnancies [17]. In two of the families, the affected pregnancies all occurred when the mothers were under 35 years of age; in the other two families, they all happened when the mothers were over 35. Low levels of T21 cells were found in the two younger mothers, 3/82 cells in blood in one case and 1/170 in blood and 3/100 in fibroblasts in the other. The authors concluded that while the multiple affected pregnancies were the result of maternal mosaicism in the younger mothers, they could have been chance occurrences in the older ones [17]. These two families where the younger mothers had three affected pregnancies are interesting because presumably once again, the cells of the ovary are likely to have had considerably higher levels of T21. Women with low level mosaicism in their ovaries seem very unlikely to produce multiple oocytes with T21 but could obviously have single affected pregnancies.

More recently, a retrospective analysis of 151 families with a DS child using both karyotyping and cytogenetic and molecular markers identified eight families with germline mosaicism [21]. In all eight cases, the mother was less than 35 years old. The prevalence of germline mosaicism in couples with a DS child in this group was thus estimated as 5.3%.

**Fig. 1:**
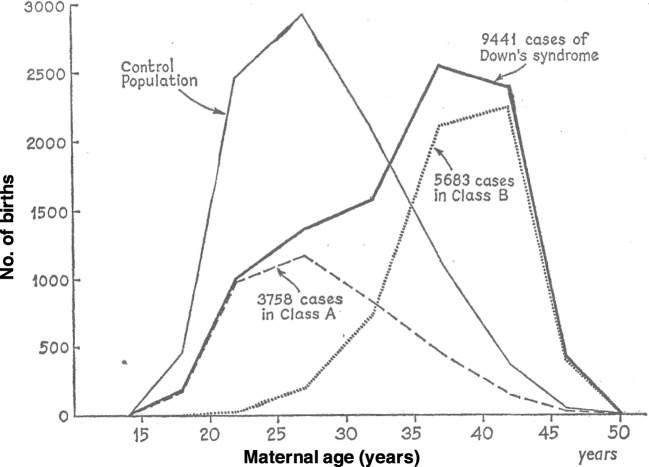
Maternal age distribution curve of 9441 cases of Down’s syndrome with control population. This was described as bitangential with the outline of a crouching sphinx. Class A cases occur independently of maternal age; this curve follows that for the control population. Class B cases are dependent on the increasing age of the mother. Figure reproduced with permission [23]

More than 40 years earlier, a seminal analysis by the great geneticist Lionel Penrose and colleagues had provided a clear rationale for such a finding. This is the only published large body of postnatal data that is pertinent to the analysis of the incidence of parental mosaicism and premeiotic aneuploidy. Following the identification of an extra small acrocentric chromosome (centromere close to one end of the chromosome) in DS cases, by Lejeune in 1959, in the early 1960s, a large body of data had been collected worldwide on cases that had been karyotyped [22]. The strong association of increasing incidence with advanced maternal age was confirmed but also the occurrence of a significant proportion of cases that were born to younger women. Detailed analysis of data from over 9000 cases from 11 countries showed that the distribution curve of maternal age for DS cases did not mirror that for the population as a whole (which peaked at about 28 years) but showed an unevenly increasing incidence that was bimodal or bitangential, described as “having the appearance of a crouching sphinx” (Fig. 1) [24]. This was taken to indicate the presence of two overlapping distributions, called classes A and B. Class A matched the pattern for the control population of all births, with a peak for maternal age at about 28 years and therefore included all cases of DS that had occurred independently of maternal age, whereas class B included the remainder where the incidence rose dramatically with increasing age of the mother. Of the 9441 cases for which data was available, 3758 were assigned to class A (almost 40%) and 5683 to class B. The proportions were determined by assuming that the youngest maternal age group (15–19 years) includes no age-dependent cases [24]. The exact proportion assigned to classes A and B in each country depends upon the general maternal age structure for all births at that time. There are various possible causes of maternal age independent aneuploidy; these are discussed in the next section. Age-related aneuploidy of maternal origin has been well studied with several possible causes identified, but little attention has been devoted to the possible contribution of age-independent factors [25].

Interestingly, corroborative data for the bimodal distribution of aneuploidy in general with respect to the mother’s age was recently obtained from the results of comprehensive chromosome screening carried out on trophectoderm biopsies from embryos at the blastocyst stage of development. A review of over 15,000 consecutive biopsies confirmed once again the close relationship between the increasing incidence of aneuploidy with maternal age above 37 years [26]. An unexpected finding was that the aneuploidy rate for the group aged between 22 and 25 years was comparable to that for mothers aged 37, both being around 40% of embryos tested [26].

### Possible causes of cases of autosomal trisomy such as DS that are age independent

The possible causes of trisomic cases that occur independently of maternal age may be (i) secondary nondisjunction, (ii) translocation or other anomaly of the critical chromosome in a parent, (iii) genes which influence nondisjunction, (iv) environmental influences. Of these, only secondary nondisjunction as a result of aneuploidy in the primary oocyte will directly lead to premeiotic aneuploidy.

Primary nondisjunction is the failure of a pair of homologous chromosomes to separate at meiosis I. Secondary or inevitable nondisjunction occurs in an oocyte that enters meiosis with an extra chromosome; one product of the first meiotic division will certainly be disomic instead of monosomic for that chromosome. Mothers with constitutional (cytogenetic anomaly that is present in all body cells), i.e. full, trisomy are very rare but those with mosaicism affecting the ovaries were estimated by Penrose and Smith [24] to account for 10% of DS cases, based upon dermatoglyphic (palm print) analysis at that time. (dermatoglyphics is a study of fingerprints, lines, mounts and shapes of the hand that is affected by genetic anomalies).

The carriers of chromosomal rearrangements involving chromosome 21 are predisposed to the production of oocytes during meiosis that have extra or missing copies of that chromosome; this occurs independently of the age of the mother. These cases include Robertsonian or reciprocal translocations, inversion of chromosome 21 or polymorphisms that could interfere with pairing. Such considerations also apply to cases of DS that have de novo rearrangements. Taken together, this group accounts for up to 5% of DS cases [24].

Another cause of age-independent trisomic cases would be variation in genes that predispose to nondisjunction at meiosis. These are not easy to identify in humans, but one factor that has been shown to be of importance in fertility is the recombination rate at meiosis; reduction in crossing over is associated with an increase in aneuploid oocytes and lower fertility [27, 28]. Proteins that have been shown to influence meiosis include PRDM9 (affects recombination), Separase and Shugoshin 1 that are involved in separation of chromosomes and chromatids [29–31].

Lastly, environmental exposure to agents such as X-rays [32, 33] would be expected to act independently of the age of the mother, although the damage inflicted could be influenced by genetic factors (reviewed in [34]). Recently, a strong link has been identified between environmental exposure to a plasticiser called as bisphenol A (BPA), to which humans are exposed on a daily basis. This is known to be an endocrine-disrupting chemical that is linked to inducing aneuploidy during meiosis in mice [35]. Moreover, a study of in vitro exposed cultured fetal ovarian tissues to BPA was suggested to have in utero implications for human females particularly during the critical events of meiotic prophase, such as pairing synapsis and recombination processes, as well as oocyte survival [36].

### Cytological proof of gonadal mosaicism

The first cytological proof of gonadal mosaicism for trisomy 21 was obtained in the case of a chromosomally normal couple that had three conceptions with trisomy 21 and one normal child [3]. The couple was referred for preimplantation genetic diagnosis allowing the chromosome 21 constitution of preimplantation embryos and unfertilised metaphase II (MII) oocytes to be assessed by means of FISH. Of the seven embryos tested, four had trisomy 21. Additionally, four unfertilised meiosis II oocytes were also assessed, of which three had an abnormal chromosome 21 constitution, i.e. either an extra chromosome 21 or an extra chromatid 21. Crucially, in one case, an extra chromatid was detected in both the MII oocyte and in the first polar body (PB1) providing the first direct evidence of a premeiotic maternal trisomic germ cell line and showing that the extra chromosome 21 can precociously divide into two chromatids prior to metaphase I (MI) of meiosis [3]. This mechanism of precocious division of the single extra chromosome greatly increases the risk of a trisomic conception, over and above the 50% risk when the single chromosome migrates as an entity to either the MII oocyte or the PB1 [3].

### Studies on oocytes

In the most recent, large-scale karyotyping study of 1397 oocytes from 792 patients (mean age 33.7 ± 4.7 years), numerical abnormalities were detected in 20.1% of samples overall. There was a clear maternal age effect, with an average of 10% of oocytes affected in the 20–25 age group versus 50–100% at ages 40–46 [37]. Numerical abnormalities caused by extra or missing chromatids were more common than aneuploidy of whole chromosomes, confirming the hypothesis put forward by Angell [37, 38]. This stated that premature separation of sister chromatids (PSSC) prior to anaphase I of meiosis would lead to random segregation and consequent nondisjunction of single chromatids. The prevalence of single chromatid anomalies compared with those affecting whole chromosomes, particularly in oocytes from older women, has been confirmed in recent studies [37, 39, 40].

Results of the application of FISH for the study of overall oocyte aneuploidy incidentally allowed for the first time detection of premeiotic aneuploidy, where the cytogenetic analysis of both the MII oocyte and the corresponding PB1 was included. FISH applied to oocyte chromatin provides accurate direct results due to the contracted nature of the DNA. Using FISH probes to investigate aneuploidy in up to nine chromosomes, three independent studies incidentally obtained evidence for germinal mosaicism with incidences of 5, 20 and 9.5%, respectively [41–43]. The determination of the origin of the aneuploidy (meiosis I or premeiotic) after chromosome copy number analysis in oocytes at different stages of maturation is explained in Table 1. The study by Pujol *et al.* is particularly interesting; this was set up to assess the accuracy of PB1 analysis in the detection of oocyte aneuploidy; the finding of evidence for germinal mosaicism was entirely incidental. In 54 MII-PB1 (metaphase II-first polar body) doublets, they observed 11 instances of additional chromosomes or chromatids without reciprocal loss, indicating a trisomic primary oocyte. Three of the 11 MII-PB1 doublets, affected with a mixture of chromosomes, were from a single patient who was aged 31 [42]. Patient variability, which may be genetically determined, is an obvious factor contributing to the range of frequencies observed in these studies. The authors considered that it was most likely that the non-reciprocal gains were the result of errors occurring during the multiple mitotic divisions in the oogonia.

**Table 1:** Interpretation of the origin of the aneuploidy (meiosis I or premeiotic) after chromosome copy number analysis in oocytes at different stages of maturation

Stage of oocyte maturation	Expected chromosome copy number	Origin of error after chromosome copy number analysis (meiosis I or premeiotic)
Germinal vesicle (GV)	Diploid (*n* = 46)	Gain or loss of any chromosome indicates premeiotic aneuploidy.
Metaphase I (MI) oocyte	Diploid (*n* = 46)	Gain or loss of any chromosome indicates premeiotic aneuploidy.
MII and PB1 analysed separately	MII–haploid (*n* = 23) PB1–haploid (*n* = 23)	Reciprocal gain or loss of any chromosome indicates meiosis I error, i.e. loss in one body should be mirrored by gain in the other.
		Non reciprocal gain of any chromosome indicates premeiotic aneuploidy.
		Non reciprocal loss may indicate premeiotic aneuploidy or loss by anaphase lag.
MII and PB1 analysed together	Diploid (*n* = 46)	Gain or loss indicates premeiotic aneuploidy.

Another informative study employing FISH technology was carried out by Costa and Wilton comparing the incidence of aneuploidies arising at meiosis with those of premeiotic origin in mature and immature human oocytes, respectively [44]. Analysis of 203 MII oocytes and 93 germinal vesicles (GV) was performed by FISH for four chromosomes (chromosomes 11, 17, 16, 22). A GV stage oocyte is a primary immature oocyte characterised by a nucleus called the germinal vesicle which is arrested in the prophase stage of meiosis I. The aneuploidy seen at the GV stage is indicative of premeiotic errors, while that seen in MII oocytes is assumed to be the product of meiosis I errors. Almost 20% of GVs, compared to 45% of MII oocytes, showed aneuploidy of more than one chromosome tested. While the aneuploidy was significantly greater in the MII oocytes, the authors state that “importantly it has also shown that a significant number of oocytes are aneuploid even prior to metaphase I.” [44].

The advent of metaphase CGH applied to single cells following whole genome amplification (WGA) enabled information on the complete chromosome complement to be obtained by molecular means for the first time [6, 7]. In one study of overall oocyte aneuploidy employing this approach, of eight informative (MII plus PB1) aneuploid oocytes, one (12.5%) provided evidence for germinal mosaicism for chromosome 13 in a 32-year-old patient [45]. In a second study of 84 MII-PB1 complexes, the overall aneuploidy rate was 32%. Fifteen (17.8%) showed the expected complementary (reciprocal) events in the two products but the remaining 13 (15.5%) events were non-reciprocal with loss or gain in the MII or PB1 but a euploid profile in the corresponding PB1 or MII, indicating an aneuploid primary oocyte [4].

A more recent study by the same Spanish group analysed 157 immature oocytes (GV or MI stage) from both IVF patients and IVF donors by metaphase CGH. Fifty-six women of average age 32.5 years included 32 IVF patients (range 25–45 years) and 24 oocyte donors (range 18–33 years) [46]. Overall, 24 oocytes of the 157 showed premeiotic aneuploidy of whole chromosomes (15.3%). Moreover, 33.9% (19/56) of women produced aneuploid oocytes. A total of 83 oocytes were from 32 IVF patients and 74 oocytes from 24 donors. The aneuploidy rate in the patient group was 15.7% (13/83) versus 14.9% (11/74) in the donor group. Premeiotic events were seen in immature oocytes from 12/32 IVF women and 7/24 donor patients [46].

**Fig. 2:**
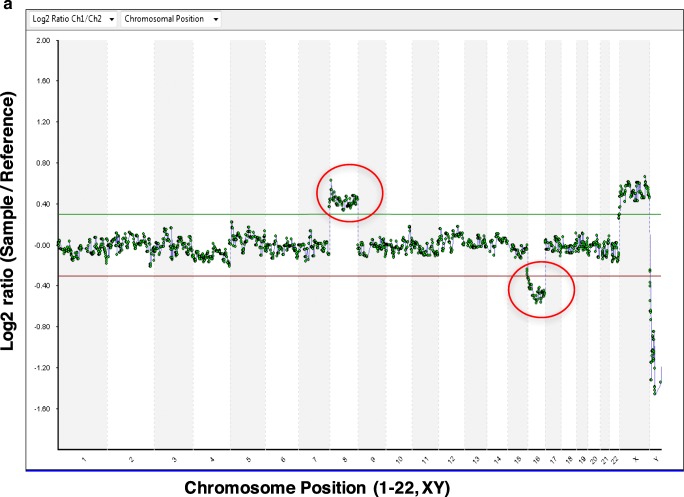

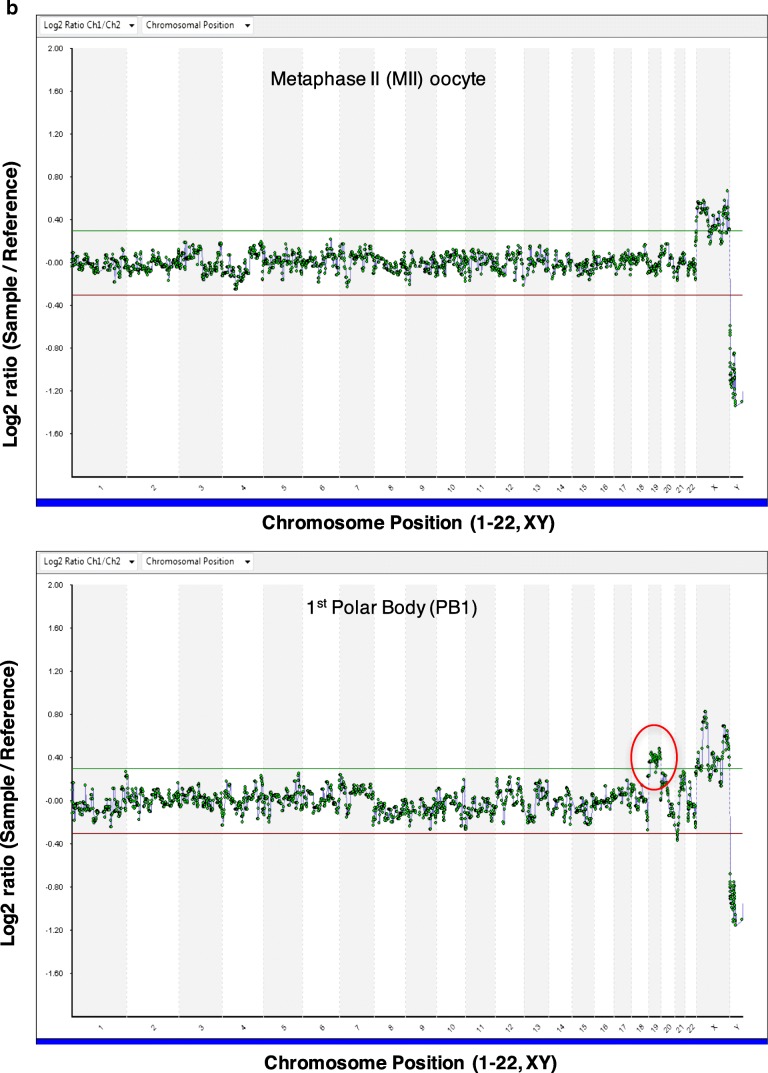
**a** aCGH profile of an aneuploid MI oocyte. The MI oocyte shows a gain of chromosome 8 and a loss of chromosome 16, both premeiotic errors. All the autosomes (except chromosomes 8 and 16) are within the thresholds of normality. **b** aCGH profile of an aneuploid MII-PB1 complex with a non-reciprocal error. The MII oocyte is euploid whereas the corresponding first polar body (PB1) shows a gain of chromosome 19

Specifically, to investigate the frequency of germinal mosaicism, a study was set up that employed microarray CGH in the analysis of oocytes unexposed to sperm [5]. All protocols used in this study were previously validated for diagnostic use. Array-CGH (aCGH) using BlueGnome 24Sure arrays was initially validated on 35 single cells from cell lines with known karyotypes. Validation results obtained had a 100% concordance rate [47]. In addition, 101 samples were monitored in follow-up analysis of diagnostic cases of which 100 agreed entirely with the diagnostic result. The research material consisted of GVs, MI oocytes and MII–PB1 complexes, unexposed to sperm; all of which are informative regarding premeiotic aneuploidy. GV’s and MI’s are expected to be diploid, MII oocytes and PB1s should each be haploid; loss in one body should be mirrored by reciprocal gain in the other. Any deviation from the expected situation provides evidence for premeiotic aneuploidy. Eighty-one informative oocytes (18 GV, 29 MI, 34 MII-PB1) from 57 women of average age 35 (29–45) years gave results. The 18 GVs were all euploid while four of 29 MI oocytes were aneuploid (Fig. 2a). Of the 34 MII-PB1 complexes, 29 were analysed after separation of the two bodies and 5 complexes were analysed where both MII and PB1 were analysed in a single reaction. Of the 29 MII–PB1 complexes separately analysed, 19 complexes showed aneuploidy; 13 complexes with reciprocal errors, as expected, and six with non-reciprocal errors, indicating premeiotic aneuploidy (Fig. 2b). Five non-separated complexes were all euploid indicating no premeiotic aneuploidy. An additional 21 samples were MIIs or PB1s alone, so the origin of the aneuploidy could not be determined, six of these were aneuploid, giving an overall aneuploidy rate of 28% (29/102) for the total 102 oocytes (18 GV, 29 MI, 34 MII-PB1, 19 MII’s only, 2 PB1’s only). In all, of the 81 oocytes that gave information regarding premeiotic aneuploidy, eight oocytes (4 MI’s and 4 MII–PB1 complexes with non-reciprocal gains) from 7 women were definitely positive for germinal mosaicism. The remaining two MII–PB1 complexes with non-reciprocal errors showed losses in one of the components, but euploidy in the other; this could be due to germinal mosaicism but also could be caused by anaphase lag in meiosis I, particularly following premature chromatid separation. Thus, in our data set, a maximum of 12.5% (10 oocytes composed of 4 MI’s, 6 MII-PB1 complexes with non-reciprocal losses/gains) were aneuploid because of germinal mosaicism, that is, premeiotic aneuploidy [5].

Taken together, data from the CGH oocyte studies suggest that the incidence of premeiotic aneuploidy lies between 10 and 20% of tested oocytes in this population of women most of whom are undergoing IVF for various reasons but are not necessarily infertile. Most of the women were undergoing routine IVF treatment but some were having preimplantation genetic testing of their embryos; no details are available for the majority of those showing germinal mosaicism. Table 2 provides a summary of the studies of oocytes that have detected premeiotic aneuploidy. The details of the number and types of oocytes examined, method of chromosome analysis, chromosomes involved in premeiotic aneuploidy and maternal ages of the women are listed.

**Table 2:** Detailed summary of studies of oocytes that have detected premeiotic anomalies included in this review

References	No. of oocytes examined that were informative for premeiotic errors	Stage of meiotic maturation	Analysis method	No. of oocytes/oocytes-PB1 complexes indicative premeiotic anomalies (%)	Chromosomes involved premeiotic anomalies	No. of patients with oocytes showing premeiotic abnormalities (mean age in years)
Mahmood et al. 2000 [41]	57 unfertilised or non-inseminated	MII-PB1 complexes	FISH chromosomes analysed: 1, 9, 13, 16, 18, 21 and X	3 (5.2%)	13 and 21	2 (ages 26 and 31)
Costa and Wilton 2000 [44]	93 immature oocytes	GVs	FISH chromosomes analysed: 11, 17 (1st round), 16, 22 (2nd round)	18 (19.3%)	11, 17, 16, 22	Not available
Pujol et al. 2003 [42]	54 IVM (non-inseminated) and unfertilised	MII-PB1 complexes	FISH chromosomes analysed: 1, 13, 15, 16, 17, 18, 21, 22 and X	11 (20%)	1,13,15,16,17,21,22 and X	9 mean age 33 (range 31-38)
^a^Gutierrez-Mateo et al. 2004 [43]	42 IVM (non-inseminated) and unfertilised	MII-PB1 complexes	CGH on PB1 aFISH on MII oocytes	4 (9.5%)	4, 15, 16, 18, X	4 mean age 39 (range 36–42)
Fragouli et al. 2006 [45]	8 IVM (non-inseminated)	MII-PB1 complexes	mCGH	1 (12.5%)	13	1 (age 32)
Obradors et al. 2010[4]	84 fresh (non-inseminated) and IVM	MII-PB1 complexes	mCGH	13 (15.5%)	2, 3, 8, 10, 11, 13, 16, 17, 19, 20, 22	12 mean age 25 (range 19–32)
Daina et al. 2014 [46]	157 immature oocytes	GVs or MIs	mCGH	24 (15.3%)	All chromosomes except 14 (highest aneuploidy rate for 21, 13, 16, 19 and 22)	19 mean age 32.5 (range 18–45)
Ghevaria et al. 2014 [5]	81 immature and fresh (non-inseminated)	GVs, MIs, MII-PB1 complexes	aCGH	10 (12.5%)	1, 2, 4, 6, 8, 12, 15, 16, 19, 22, X	9 mean age 34 (range 23–40)

IVM (in vitro matured oocytes): these were immature oocytes discarded from IVF cycles and were matured in vitro

Unfertilised oocytes: MII oocytes that failed to fertilise after IVF or ICSI

Non-inseminated: MII oocytes unexposed to sperm.

Fresh: oocytes retrieved at MII stage displaying a PB1

GVs: oocytes at germinal vesicle stage

MIs: oocytes at metaphase I stage

MIIs: oocytes at metaphase II stage

PB1–1^st^ Polar Body

^a^Gutierrez-Mateo et al. 2004 [43]—the PB1s were analysed by mCGH (metaphase CGH). The MII oocytes were analysed by FISH (probe selection was based on aneuploidy seen in PB1). Three sets of FISH probes used as follows: set A: 13, 16, 18, 21, 22; set B: X, 1, 15, 17; set C: 3, 4, 6, 7, 8, 9, 10, 14, 19

### Direct investigation of ovarian cells

Gonadal mosaicism may also be investigated using the alternative cytological approach of direct analysis of fetal ovarian tissue [48]. In this study, ovarian cells from eight female fetuses between 14- and 22-weeks gestation were studied. The fetuses were phenotypically normal and were the products of social terminations of pregnancy. Meiotic, premeiotic and mesenchymal stromal cells were subject to analysis by FISH using two different fluorescent probes for chromosome 21 to determine the number of copies present. The number of cells studied in each case was between 967 and 2200. Trisomy 21 mosaicism was detected in every sample, varying between 0.2 and 0.88% (average 0.54%). Meiotic, premeiotic and stromal cells were equally affected. If this frequency of mosaicism applied equally to all chromosome pairs, then the overall frequency would be 11.5%, within the range found with comprehensive chromosome analysis by CGH. An interesting corollary to this finding in female fetal gonads is that the same is not true of the male germ cells. Testicular cell nuclei from four normal male fetuses (14–19 weeks) were investigated by FISH analysis of chromosome 21 using the same dual probe strategy [49]. At least 2000 cells were analysed per case but not a single example of trisomy 21 mosaicism was detected [49]. It was suggested that the difference is likely to be due to the much more stringent system of cell cycle control that operates during meiosis in the male compared with that of the female mammal [50]. Testicular cells with an additional chromosome are likely to be halted in development and to undergo apoptosis. A subsequent study in oocytes by Morris and colleagues [51], obtained quite different results, failing to confirm the incidence of trisomy found by the Hultén group [48]. Morris and colleagues analysed 51,146 cells from eight fetal samples, although they found a small number of trisomic cells (13 in all—0.025%), in the analysis of both ovarian and skin samples, they failed to find a single example of trisomy 21. They used a different type of fluorescent probes, ‘break apart probes’, but perhaps more significantly, the fetal samples were of 10–14-week gestation compared to 14–22 weeks in the study by Hultén and colleagues in 2008 [48]. Hultén and co-workers subsequently demonstrated a highly significant difference in the incidence of cells with trisomy 21 between the first and second trimesters of pregnancy, suggesting that an accumulation of these cells takes place as oogenesis progresses [52]. One criticism of these studies was that no attempt was made to determine in which stage of meiotic prophase the analysed oocytes fell [53]. Following initial analysis of cells from two fetuses with trisomy 21 that showed leptotene to be the preferable stage for detection of three distinct signals, this group confined their analysis to this stage of prophase in oocytes from a further seven second trimester female fetuses that were presumed to be chromosomally normal [53]. This approach inevitably reduced the number of cells available for analysis; from seven samples, a total of 1206 leptotene stage cells were analysed with a FISH probe that detected centromeres of both chromosomes 13 and 21, together with one that detected chromosome 16. Not a single example of true trisomy was noted. However, two criticisms could be levelled at this study compared with that by Hulten and team in 2008 [48]; firstly that only one probe was used for chromosome 21, and that is one that is not considered to be very efficient, and secondly that the average number of cells analysed was less that 200 per sample. Since the average incidence of trisomic prophase cells was 1 in 200 in the Hulten’s 2008 [48] study, the Rowsey [53] results cannot be said to rule out the existence of a small proportion of cells in meiotic prophase that are trisomic. In summary, direct analyses of ovarian cells have provided variable results with regard to germinal mosaicism.

### Comprehensive chromosome analysis of the three products of female meiosis

An innovative approach involving aCGH analysis of the three products of meiosis, 1st PB (PB1), 2nd PB (PB2) and corresponding one cell embryo (zygote) (referred to as zygote–PB trios) was employed in the study of meiotic errors caused by predivison of chromatids in women of advanced maternal age [39]. The copy number changes for all chromosomes were analysed for 105 zygote–PB trios. The results were recorded as gain (G), loss (L) or normal (N) for each chromosome copy number change as a three-letter code representing the copy number in PB1/PB2/zygote, respectively. Array results recognised predicted segregation patterns for errors occurring in maternal meiosis (I and II) by either whole chromosome nondisjunction or predivision of sister chromatids. The predicted segregation patterns included:

- LGG, GLL (meiosis I, non-disjunction),
- LNG, GNL (meiosis I, predivision),
- GLN, LGN (meiosis I premature predivision of chromatids balanced at meiosis II),
- LLG, LGL (meiosis I and meiosis II error),
- NGL, NLG (meiosis II non-disjunction or predivision).

Additionally, errors were categorised as possibly introduced by the fertilising sperm or caused by early chromosome loss before the first mitotic division; these being NNG (paternal trisomy), NNL (paternal monosomy/chromosome loss). Apart from these recognised patterns, other patterns were also detected such as NGN, NLN, NLL, GNG, GNN, GGN, LNN, LLN. In their study, segregation pattern analysis showed that of the 353 chromosome errors detected, 275 (78%) of these were in accordance with the predicted patterns. The remaining 78 did not follow any of these recognised patterns of error [39]. The authors dismissed the possibility that these unrecognised patterns could be due to germinal mosaicism, but closer analysis performed for this review reveals that this could be the cause at least in some cases.

**Fig. 3:**
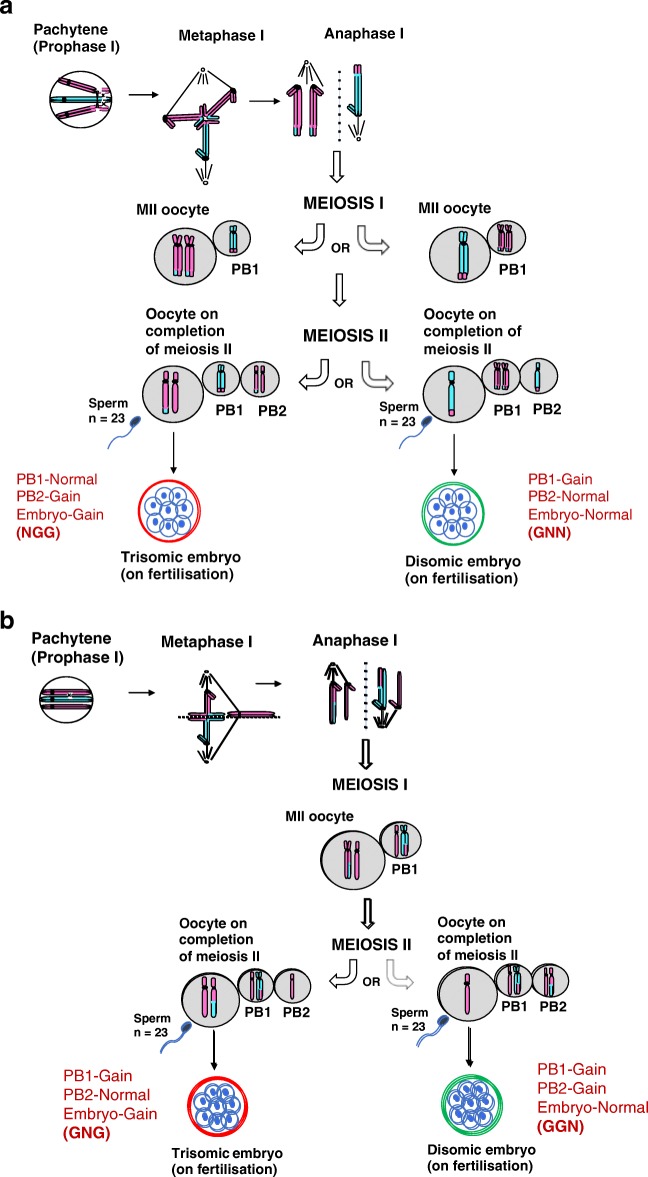

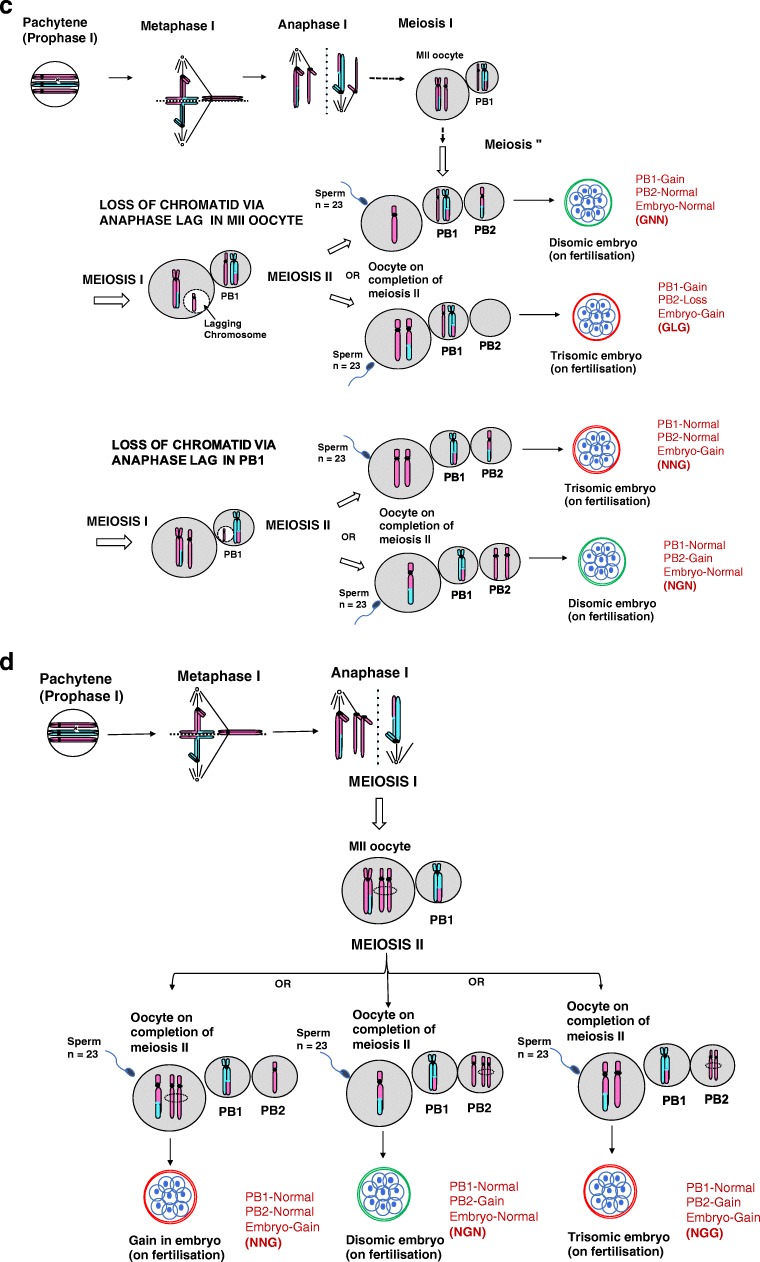
**a** Segregation patterns detected by aCGH if the primary oocyte is trisomic with formation of a trivalent at prophase of meiosis I. Figure adapted with permission from [48]. **b** Segregation patterns detected by aCGH if the primary oocyte is trisomic with formation of a bivalent and univalent at meiosis I and the univalent separates into two chromatids that segregate independently. Figure adapted with permission from [48]. **c** Segregation patterns detected by aCGH if the primary oocyte is trisomic with formation of a bivalent and univalent at prophase of meiosis I, in the event of loss of a chromatid via anaphase lag in meiosis I either from the MII oocyte or from PB1. Figure adapted with permission from [48]. **d** Segregation patterns detected by aCGH if the primary oocyte is trisomic with formation of a bivalent and univalent at prophase of meiosis I in the event of the premature separation of the univalent into two chromatids that segregate together to the MII oocyte. Figure adapted with permission from [48]

Additional analysis has been performed for the purpose of this review in order to identify the various possibilities for the copy number changes that could be predicted (by aCGH) if during meiosis, the primary oocyte is trisomic because of germinal mosaicism (assuming there were no parental constitutional aneuploidies for that chromosome) and would be as follows:

1. *Trivalent pairing*: If during prophase of the first division of meiosis, the three chromosomes align and pair as a trivalent, and two of the recombined chromosomes pass to the MII oocyte and one to the PB1, on completion of the second meiotic division (meiosis II), the pattern seen would be NGG. However if one chromosome passes to the MII oocyte and two to the PB1, on completion of meiosis II, the pattern seen would be GNN (Fig. 3a)
2. *Bivalent plus univalent pairing*: In addition to the above, if during prophase of meiosis I, the chromosomes align and pair as a bivalent plus univalent, the single chromosome could separate into two chromatids and one pass to each of the PB1 and the MII oocyte, the patterns seen would be GNG or GGN (Fig. 3b)
3. *Bivalent plus univalent pairing with anaphase lag*: At this stage, in the case where one chromatid is lost from MII oocyte via anaphase lag, it would lead to GNN or GLG or if lost from the PB1 via anaphase lag, the two possibilities would be NNG or NGN (Fig. 3c).
4. *Bivalent plus univalent pairing with premature univalent separation*: If the univalent separates prematurely into two chromatids during meiosis I, and the two separated chromatids stay together in the oocyte, various alternative outcomes are possible depending on the outcome of meiosis II. The patterns seen could be NNG, NGN or NGG (Fig. 3d). However, if the two separated chromatids stay together in the PB1, the pattern seen would be GNN.

We have carefully re-analysed the data presented in the supplementary information of the paper by Handyside and colleagues [39]. The number of oocytes with these unrecognised patterns that are consistent with germinal mosaicism has been tabulated (Table 3). Of these patterns consistent with germinal mosaicism, the number of oocytes with the most common segregation patterns were GNN, NGN and NNG (Table 3). For GNN, 9 trios with 17 copy number changes were identified, for NGN, another 9 trios with 11 copy number changes were seen and for NNG 11 trios with 18 copy number changes; additionally, a single example each of GNG and GGN was found. In their analysis, Handyside and colleagues consider the segregation result of NNG to be trisomy in the zygote due to a paternal error [39]. However, FISH analysis of all chromosome errors in spermatozoa leads to an overall incidence of aneuploidy of 2.26% [54]. Thus, in the 105 trios analysed by Handyside and colleagues, only about three errors due to paternal disomy would be expected in the zygotes, not 18 as seen, several of them multiples in the same trio. In all, in 27 out of the 105 (26%) trios, copy number changes are consistent with the situation where the primary oocyte entered meiosis with a trisomic chromosome complement for at least one chromosome (Table 3). Moreover, this would be a minimal estimate since other possible scenarios have not been considered, including germinal mosaicism for monosomy.

**Table 3:** Segregation patterns expected when the primary oocyte is trisomic due to a premeiotic error (analysis based on the data presented by Handyside and colleagues) [39]

Segregation patterns if oocyte is trisomic due to a premeiotic error	No. of oocytes with the particular segregation pattern	No. of copy number changes associated with this pattern
Trivalent formation		
NGG	0	0
GNN^a^	9	17
Bivalent + univalent formation		
GNG	1	1
GGN	1	1
NNG^b^	11 (7^c^)	18
NGN^b^	10 (9^c^)	11
Total	32	48
Percentage of oocytes with at least one segregation pattern consistent with premeiotic aneuploidy	25.7% (27/105)	

The three-letter code represents the copy number in PB1/PB2/zygote, respectively *N* normal, *G* gain

^a^At the time of bivalent plus univalent pairing of the chromosomes during meiosis in a trisomic primary oocyte, loss of one chromatid in MII oocyte via anaphase lag would also lead to patterns of GNN (refer to Fig. 3c).

^b^Loss of one chromatid in PB1 via anaphase lag would also lead to patterns of NNG and NGN

^c^Number of zygote-PB trios with NNG or NGN as sole segregation pattern due to premeiotic error

A more recent paper performed analysis via single-nucleotide polymorphism (SNP)-array and maternal haplotyping on 23 trios (13 activated oocyte-PB trios and 10 embryo-PB trios) and thereby generated data on crossover patterns and recombination rates by producing high-resolution MeioMaps through the analysis of all three distinct products of meiosis. This was carried out by whole-genome amplification of the oocytes, polar bodies and maternal genomic DNA, genotyped at ~ 300,000 SNP loci, along the entire lengths of all chromosomes [28]. For the 529 chromosome pairs assessed for aneuploidy in the trios, the authors state that “all gains and losses seen were reciprocal and so do not support germline mosaicism as a major factor in the maternal age related increase in aneuploidy in humans”. Additional analyses by the authors for the purposes of this review were performed assessing the possibility of detection of the various segregation patterns after SNP genotyping and meiomapping, arising due to germinal mosaicism. Close comparison of the segregation patterns detectable by aCGH when the primary oocyte is trisomic with the patterns seen by SNP analysis in such a situation shows that not all possible permutations are detectable by SNP analysis alone and that the detection of germinal mosaicism requires copy number as well as SNP analysis for accurate detection. SNP analysis alone cannot detect copy number changes if some of the chromatids of the maternal chromosomes involved have identical haplotypes (via mitotic nondisjunction) and so will not detect cases of germinal mosaicism caused by mitotic errors during expansion of the oogonia, and this may be the most common mode of origin [42].

A scholarly study largely focused on analysis of recombination also provides information on the origin of oocyte aneuploidy by genome analysis of single nuclei using the technique of multiple annealing and looping-based amplification (MALBAC) [55]. In this case, aneuploidy could be determined by depth of coverage at a megabase resolution. Donated oocytes were obtained from eight fertile women aged 25–35 years and fertilised by intracytoplasmic sperm injection (ICSI). Their 183 samples successfully analysed were composed of ‘triads’ of 67 PB1, 64 PB2 and 52 female pronuclei (FPN). Aneuploidy was first determined by checking that the sum of chromatids (C) from PB1 (2C) + PB2 (1C) + FPN (1C) = 4 for each triad across the 23 chromosomes. PB1 is expected to have 2 sister chromatids (2C) (but the haploid complement of chromosomes, not diploid as described) whereas the PB2 and FPN are each expected to have 1C of each autosome and of the X chromosome. A total of 43 of 44 triads had 4 chromatids with the remaining one showing losses in both PB2 and the FPN with no concomitant gains, which they attribute to either failure of DNA replication or, more likely, abnormal segregation. We propose anaphase lag occurring in the oocyte as another mechanism to provide an explanation for the result obtained for the one aneuploid triad. Apart from this one instance of chromatid loss, they detected 12 aneuploid oocytes (deduction of aneuploidy in the oocytes was based on aneuploidy observed in the PB1 and PB2 and confirmed later by direct sequencing of the FPN) due to abnormal segregation. Interestingly, they observed fewer meiotic crossovers in the aneuploid oocytes [55]. In supplementary table S6 (as published in Hou et al., 2013), in seven of the 12 aneuploid oocytes, the aneuploidies are attributed to MI errors, one to an MII error and in three cases to errors in both MI and MII. However, it can be seen that in two triads [samples S0103; S0609] where the error was attributed to MI, no result was obtained from the female pronucleus (FPN) with gains/losses in the PB1 and euploid PB2, so the origin of the error was unknown. We suggest that an alternative explanation could be a trisomic primary oocyte, as shown in Fig. 3a.

So, although these two papers that employ in-depth analysis of oocyte material to obtain haplotypes provide important information concerning mechanisms that lie behind the generation of aneuploidy, the outcome of the studies cannot rule out a contribution of premeiotic aneuploidy to the overall burden of chromosomally abnormal oocytes

## Conclusion

Although FISH analysis provided the first direct cytological confirmation of the existence of germinal mosaicism leading to aneuploidy of premeiotic origin, its application is necessarily limited to certain chromosome pairs. Only the CGH data from various sources covers all chromosomes and so may be used to provide an estimate of the frequency of germinal mosaicism among oocytes either from patients undergoing IVF, but not necessarily infertile, or from oocyte donors. Four CGH studies [4, 5, 39, 46] gave an indication of the incidence from a reasonable number of samples: Obradors and colleagues [4], 15.5% of 84 informative oocytes; Handyside and colleagues [39], a maximum of 26% of 105 trios; Daina and team [46], 15.3% of 157 oocytes; and Ghevaria and colleagues [5] 2014, a 12.5% maximum from 81 informative oocytes. Pujol and colleagues noted an unusual case with three oocytes affected with premeiotic aneuploidy [42]. The existence of patients with a particular predisposition to this type of aneuploidy will clearly affect the incidence in these studies with relatively small numbers of oocytes. Overall, the studies of oocytes by comprehensive chromosome testing indicate that on average, 15% of all premeiotic oocytes are likely to be affected by germinal mosaicism that has led to premeiotic aneuploidy.

However, this gives only a partial picture of events. How does this compare with the frequency of errors that occur during meiosis I? Published data provides some insight; Costa and Wilton found that almost 20% of GV’s, compared to an assumed 45% of MII oocytes showed aneuploidy of more than 1 chromosome tested [44]. In our aCGH study of 102 oocytes with conclusive results, 29 were aneuploid and the source of the error was known in 21. In eight (38%), the origin was premeiotic, and in 13 (62%), the error occurred during meiosis I [5]. In terms of total errors (aneuploidy events), there were 12 premeiotic errors (only gains included) (40%) whereas 18 meiosis I errors (both gains and losses included) (60%) were seen. The average maternal age was 35 years. None of the women whose oocytes had premeiotic errors were infertile [5]. In comparison, for the 105 zygote-PB trios, analysed by Handyside and colleagues, 48 possible premeiosis aneuploidy events were seen (28%) which included segregation patterns in PB1/PB2/zygote of GNN, GNG, GGN, NNG and NGN. Only those segregation patterns that correspond to gains in the oocyte were included, whereas 125 meiosis I errors were seen (72%) which included segregation patterns in PB1/PB2/zygote of LGG, LNG, LLG, GLL, GNL, LGL, LGN and GLN [39]. The overall maternal age was 40 years; almost all were undergoing preimplantation genetic screening (PGS) for aneuploidy. The increased proportion of MI errors in this group compared with the other data is probably a reflection of the advanced maternal age in this group. In summary, data so far available suggests that, depending upon the maternal age, up to 40% of aneuploidy present in oocytes at the end of MI may be due to germinal mosaicism.

As with aneuploidy originating during meiosis I and II, oocytes with premeiotic errors will rarely lead to the birth of a live born infant; most will contribute to the high level of preimplantation, and prenatal, embryonic and fetal death. For this reason, the only substantial postnatal information available is the original data on DS births that proves the existence of the relatively common class that is due to causes independent of maternal age, to which premeiotic aneuploidy is a major contributor [24]. Hence, premeiotic aneuploidy is one of the age-independent mechanisms that predispose women to produce aneuploid gametes and may be linked to their sub-fertility or infertility.
